# Malignant transformation of intraductal papillary neoplasm of the bile duct following non-standardized biliary-enteric drainage for hepatolithiasis: two case reports and literature review

**DOI:** 10.3389/fonc.2026.1980694

**Published:** 2026-10-26

**Authors:** Jinpeng Peng, Pingzhou Yang, Hao Huang, Chuang Peng, Zhiguo Tan

**Affiliations:** 1 Department of Hepatobiliary Surgery, Hunan Provincial People’s Hospital/The First Affiliated Hospital of Hunan Normal University, Changsha, Hunan, China; 2 Department of Oncology, Hunan Provincial People’s Hospital/The First Affiliated Hospital of Hunan Normal University, Changsha, Hunan, China

**Keywords:** biliary-enteric drainage, case report, chronic cholangitis, hepatolithiasis, intraductal papillary neoplasm of the bile duct

## Abstract

**Background:**

Intraductal papillary neoplasm of the bile duct (IPNB) is a rare biliary epithelial neoplasm with malignant potential. It is characterized by intraductal papillary growth, variable mucin production, and a close association with chronic biliary inflammation.

**Case presentation:**

We describe two patients with hepatolithiasis who developed IPNB after non-standardized biliary-enteric drainage without complete common bile duct transection. Both patients had long-term enterobiliary reflux and chronic cholangitis. Case 1 was diagnosed with intestinal-type IPNB with high-grade intraepithelial neoplasia and focal invasive mucinous adenocarcinoma and remained recurrence-free at 12 months despite a microscopically positive ductal margin (R1 resection). Case 2 had pancreatobiliary-type IPNB with moderately to poorly differentiated invasive adenocarcinoma and developed rapid postoperative peritoneal and distant metastases.

**Conclusion:**

In these two patients with hepatolithiasis, non-standardized biliary-enteric drainage with a retained distal common bile duct and blind pouch was associated with long-term enterobiliary reflux, chronic cholangitis, and subsequent IPNB. These findings suggest a plausible contributing mechanism rather than an established causal relationship. When biliary-enteric reconstruction is otherwise indicated, surgical techniques that avoid a residual blind pouch and minimize enterobiliary reflux should be considered. Long-term surveillance may be warranted in patients with long-standing biliary inflammation after non-standardized drainage.

## Introduction

Intraductal papillary neoplasm of the bile duct (IPNB) is a grossly identifiable neoplastic lesion characterized by intraductal papillary proliferation within the biliary lumen, which can arise at any site along the intrahepatic and extrahepatic biliary tree. It is widely recognized as a premalignant biliary epithelial lesion that progresses to invasive cholangiocarcinoma through a stepwise dysplasia-carcinoma sequence (1–4).

Hepatolithiasis is an established predisposing factor for chronic cholangitis and biliary carcinogenesis, with a particularly high prevalence in East Asian populations (3–5). In patients with prior biliary-enteric drainage, incomplete separation of the bile duct from the intestinal tract may cause persistent enterobiliary reflux and biliary bacterial colonization. These pathological processes may lead to repeated injury and aberrant repair of the biliary epithelium, and may contribute to epithelial malignant transformation. Herein, we present two clinical cases to illustrate this potential carcinogenic pathway and further delineate the heterogeneous clinical behavior of IPNB in the context of chronic biliary injury.

## Case presentation

The clinicopathological characteristics of both cases are summarized in **Table 1**

**Table 1:** Clinicopathological characteristics of the two IPNB cases.

Characteristic	Case 1	Case 2
Sex/Age	Male/66 years	Female/69 years
Previous surgery	Cholecystectomy with partial left hepatectomy (2007); two afferent-loop biliary-enteric drainage procedures (2010, 2014); common bile duct not transected.	Cholecystectomy for gallstones (approximately 30 years earlier); biliary-jejunostomy for hepatolithiasis (approximately 12 years earlier).
Presenting symptoms	Recurrent right upper quadrant pain with chills, fever, and jaundice (acute cholangitis).	Dull, distending upper abdominal pain for 2 months; mild right upper quadrant tenderness.
Initial diagnosis	Recurrent hepatolithiasis with cholangitis; no definite malignant signs preoperatively.	Large right hepatic lobe mass with biliary obstruction, suspicious for malignancy.
Tumor markers	CA19-9, 170.92 U/mL. CEA and CA125: normal;	CEA, 225.96 U/mL; CA19-9, 53.52 U/mL; CA125, 66.47 U/mL.
Operation performed	Completion residual left hemihepatectomy, hilar bile duct excision, and Roux-en-Y biliary reconstruction.	Right hemihepatectomy and regional lymphadenectomy.
Tumor size (maximum dimension)	4.5 cm (measured on the residual left liver specimen: 4.5 × 2 × 1 cm)	12 cm (gross tumor: 12 × 8 × 6.5 cm).
Pathological type	Intestinal-type IPNB.	Pancreatobiliary-type IPNB.
Associated carcinoma	High-grade intraepithelial neoplasia with focal invasive mucinous adenocarcinoma.	Moderately to poorly differentiated invasive adenocarcinoma.
Immunohistochemistry	MUC5AC(+), MUC2(+), CDX2(+), CK20(+), CEA(+), CK7(+), SATB2(-), wild-type p53.	MUC5AC(+), MUC2(-), CDX2(-), CK20(-), CEA(+), CK7(+), CK19(+), wild-type p53.
Ki-67 index	60%-70%.	30%.
Resection margin	R1 (microscopically positive for non-invasive papillary tumor at ductal stump; invasive component negative)	R0 (liver resection margin negative for carcinoma)
Lymph node status	Not assessed.	Negative: 0/7 (station 12), 0/3 (station 13), and 0/6 (per-intestinal).
Follow-up status	Alive without recurrence at 12 months post-surgery, with normalized imaging and tumor markers.	Recurrence with peritoneal and distant metastases at 11 months.

### Case 1

A 66-year-old man was admitted with a 19-year history of recurrent right upper quadrant pain and a 4-day history of chills, fever, and jaundice. He had underlying hepatolithiasis. His surgical history included cholecystectomy with partial left hepatectomy in 2007 without biliary-enteric anastomosis, followed by two repeated afferent-loop biliary-enteric drainage procedures in 2010 and 2014 for recurrent stones. In both biliary reconstructions, the common bile duct was not transected, resulting in a persistent biliary blind pouch and long-term enterobiliary reflux. The original operative records were unavailable; therefore, the exact rationale for preserving the common bile duct could not be definitively determined. At that time, non-standardized afferent-loop jejunohilar anastomosis without common bile duct transection was performed in some centers for complex recurrent hepatolithiasis, particularly when hilar inflammation, adhesions, or previous surgery made formal hilar dissection technically difficult. This approach differs from current recommended practice, in which Roux-en-Y hepaticojejunostomy with complete transection of the extrahepatic bile duct and a tension-free end-to-side anastomosis is preferred when biliary-enteric reconstruction is indicated (6, 7). One month before admission, percutaneous drainage had been performed for a left hepatic abscess.Laboratory tests indicated acute cholangitis, with leukocytosis, an increased neutrophil ratio, and thrombocytopenia. Liver function tests showed hypoalbuminemia and elevated direct bilirubin, alkaline phosphatase, and γ-glutamyl transpeptidase levels, suggesting chronic biliary injury, obstructive jaundice, and malnutrition. The serum CA19–9 level was markedly elevated to 170.92 U/mL.

#### Imaging findings

Axial T2-weighted MRI (**Figures 1A, B**) demonstrated cystic and irregular tubular dilatation of the bile ducts in the residual left hepatic lobe, with irregular intraluminal hypointense filling defects. In combination with intraoperative findings, these lesions were considered to represent mixed mucinous plugs, neoplastic secretions, and hepatolithiasis rather than simple stones. Heterogeneous hepatic parenchymal signal intensity suggested long-standing cholestasis and chronic inflammation, while irregular thickening of the hilar bile ducts reflected biliary remodeling after repeated biliary-enteric anastomoses. Atrophy of the right anterior hepatic segment and residual left hepatic lobe was also observed.

**Figure 1:**
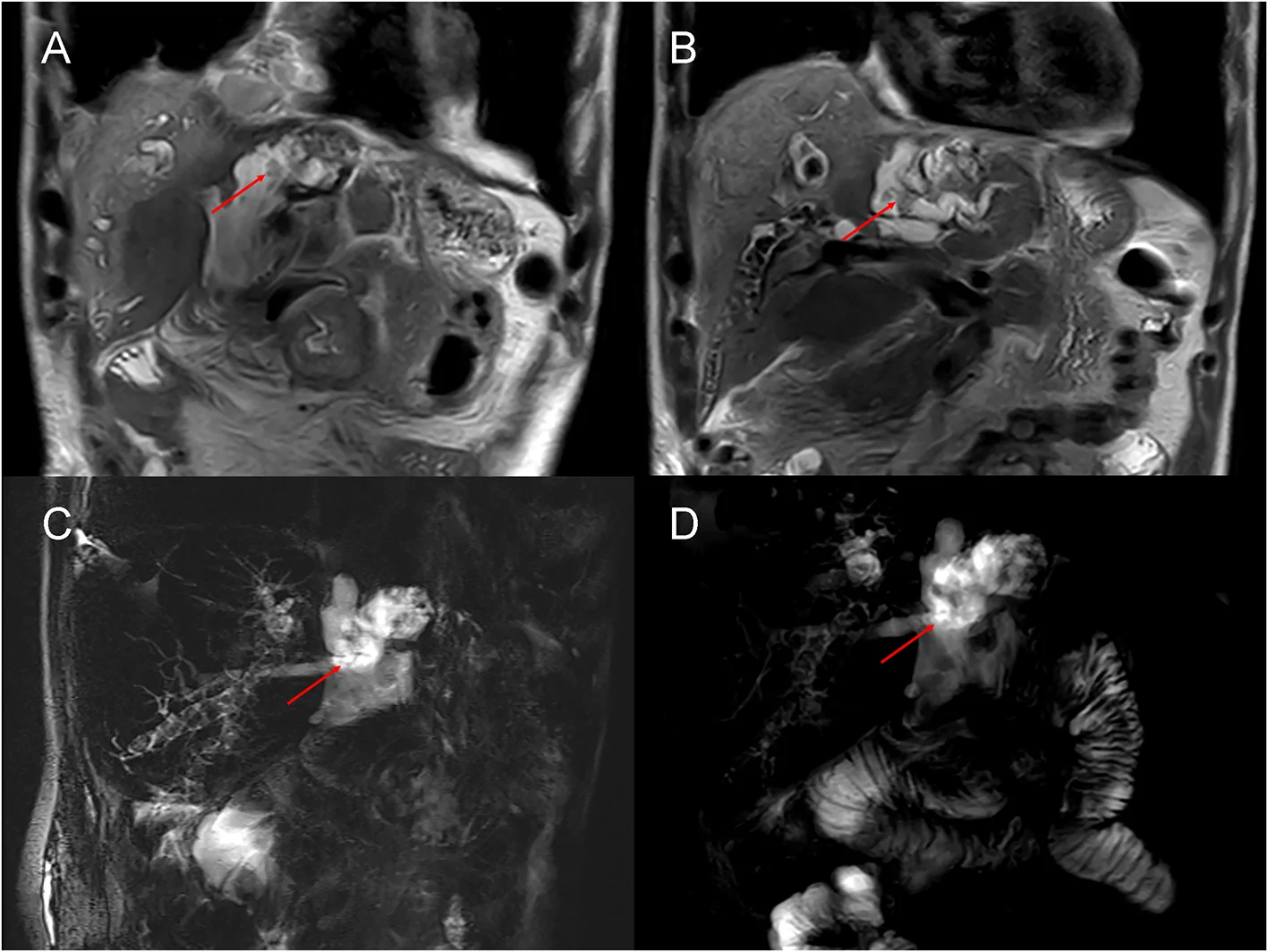
Preoperative MRI and MRCP findings in Case 1. **(A, B)** Axial T2-weighted images show dilated bile ducts with intraluminal filling defects, thickened hilar bile ducts, and atrophy of the right anterior hepatic segment and residual left hepatic lobe. **(C, D)** MRCP shows diffuse beaded biliary dilatation, multiple filling defects, and an anastomotic stricture involving the right anterior hepatic segment.

MRCP (**Figures 1C, D**) showed diffuse dendritic and beaded intrahepatic biliary dilatation with multiple filling defects. The biliary branches were distorted, narrowed, and interrupted. Abrupt ductal tapering at the hepatic hilum (**Figure 1C**) corresponded to an anastomotic stricture involving the right anterior hepatic segment. Alternating strictures and dilatations of the distal bile ducts (**Figure 1D**) were compatible with persistent enterobiliary reflux and chronic cholangitis. The gallbladder and common bile duct were not visualized on MRCP.

Preoperatively, the filling defects were suspected to represent recurrent hepatolithiasis without definite malignant features. Intraoperatively, the intraductal lesions were confirmed to be a combination of stones and diffuse papillary mucinous proliferations.

#### Intraoperative findings

An afferent-loop jejunohilar anastomosis was identified, with an untransected common bile duct and a residual blind pouch. After the anastomosis was opened, a large amount of gelatinous mucin drained from the biliary lumen, and extensive papillary tumor lesions were observed throughout the residual left intrahepatic bile ducts (**Figure 2A**). Radical resection, including completion residual left hemihepatectomy and hilar bile duct excision, was performed. The previous biliary-enteric anastomotic segment was excised, the common bile duct was completely transected, and bile flow was re-established via a Roux-en-Y hepaticojejunostomy.

**Figure 2:**
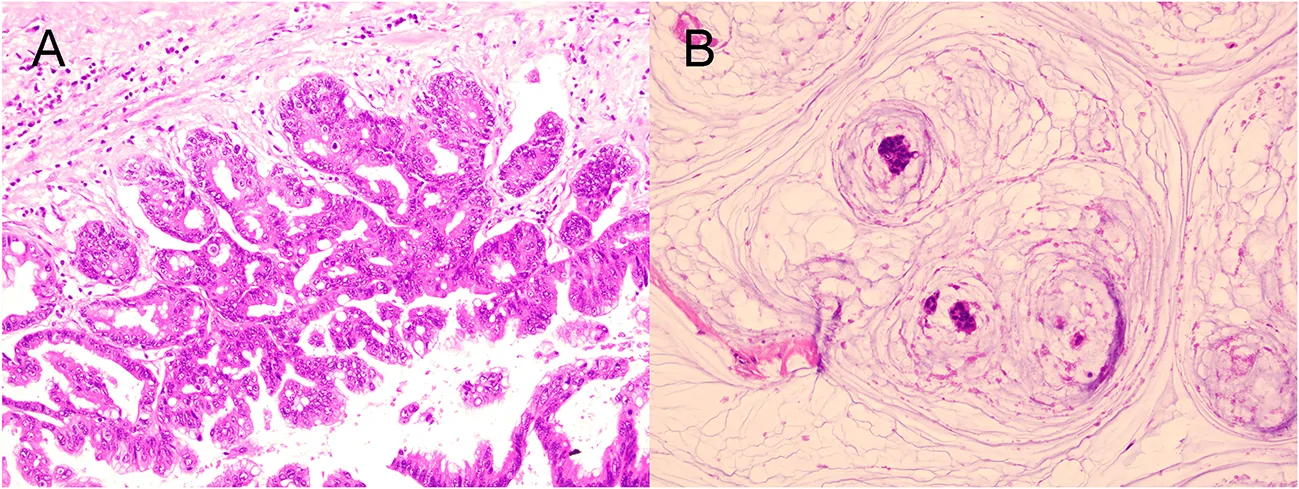
Histopathological features of intestinal-type intraductal papillary neoplasm of the bile duct (IPNB) in Case 1. **(A)** Low-power hematoxylin and eosin (H&E) staining (×100) shows complex papillary structures with fibrovascular cores lined by tall columnar epithelium, consistent with intestinal-type differentiation. **(B)** High-power H&E staining (×200) shows prominent intracytoplasmic mucin vacuoles displacing nuclei toward the cell periphery, resulting in signet-ring cell-like changes. This histological finding is concordant with the abundant gelatinous intraductal mucin observed intraoperatively.

#### Pathology

Histopathological examination confirmed intestinal-type IPNB with high-grade intraepithelial neoplasia and focal invasive mucinous adenocarcinoma. According to the original pathology report, the invasive component was described qualitatively as ‘focal’ without providing a precise diameter, which represents a limitation of the pathological data; notably, no vascular or perineural invasion was identified (**Figure 2B**). The resection margin was microscopically positive for non-invasive papillary tumor components at the ductal stump of the remnant liver (R1 resection), though the invasive foci showed no such features (**Figure 2B**). Immunohistochemically, the neoplastic cells were positive for CK7, MUC5AC, MUC2, CDX2, CK20 and CEA. SATB2 was negative. The Ki-67 labeling index ranged from 60% to 70%, and p53 staining showed a wild-type pattern (**Supplementary Figure 1A**).

#### Follow-up

Postoperative follow-up was performed regularly with serum tumor markers and abdominal imaging. At the 12-month follow-up, there was no evidence of tumor recurrence or new biliary stricture. The patient remained asymptomatic with normal liver function.

### Case 2

A 69-year-old woman was admitted with a 2-month history of dull, distending upper abdominal pain. Her surgical history included cholecystectomy for gallstones approximately 30 years earlier and biliary-jejunostomy for hepatolithiasis at Xiangya Hospital 12 years previously. Physical examination revealed mild right upper quadrant tenderness.

Laboratory tests showed hypoalbuminemia (29.08 g/L), mildly elevated transaminase levels, and markedly elevated cholangiolytic enzymes, including alkaline phosphatase (567.8 U/L), γ-glutamyl transpeptidase (303.3 U/L), and 5’-nucleotidase (117.8 U/L). Tumor markers were significantly increased: CEA, 225.96 U/mL; CA19-9, 53.52 U/mL; and CA125, 66.47 U/mL. Direct bilirubin was slightly elevated at 7.32 μmol/L.

#### Imaging findings

Contrast-enhanced CT demonstrated a large heterogeneous mass in the right hepatic lobe with invasion of the hepatic capsule. The lesion showed progressive enhancement across imaging phases, suggesting a fibrotic and infiltrative tumor component. Diffuse intrahepatic biliary dilatation was also observed, consistent with biliary obstruction secondary to an intraductal mass-forming lesion (**Figure 3A**).

**Figure 3:**
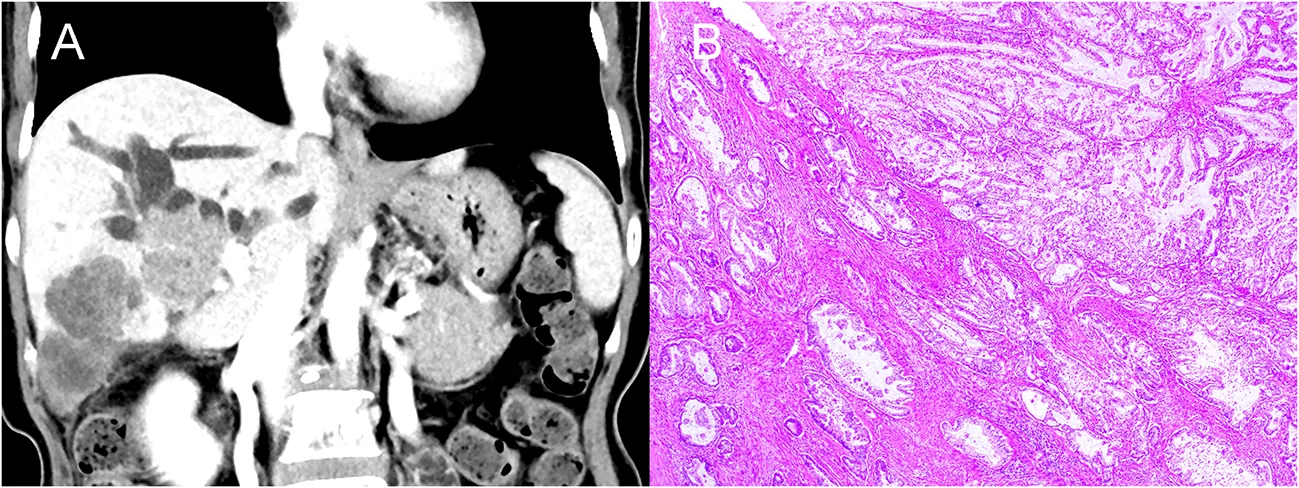
Contrast-enhanced abdominal CT findings in Case 2. **(A)** Coronal reconstruction shows a large mass in the right hepatic lobe involving the hepatic hilum, accompanied by diffuse intrahepatic biliary dilatation. **(B)** H&E staining (×40) shows irregular cystic and tubular dilatation of bile ducts filled with abundant pale, amorphous mucin. The bile duct wall shows fibrous stromal proliferation, and papillary tumor fronds lined by pancreatobiliary-type mucinous epithelium protrude into the mucin-filled lumen. Focal epithelial breakthrough into the fibrous stroma indicates microinvasion.

#### Intraoperative findings

Intraoperative exploration showed compensatory hypertrophy of the left hepatic lobe and atrophy of the right hepatic lobe. A hard mass was palpable in the right liver adjacent to the intrahepatic bile ducts. The patient had a pre-colic afferent-loop jejunohilar anastomosis without transection of the common bile duct. The blind afferent limb was markedly dilated (approximately 20 cm), with torsion, distal stricture, and severe adhesions (**Supplementary Figure 2**). Right hemihepatectomy with regional lymphadenectomy was performed. Gross examination of the resected liver showed dilated right intrahepatic bile ducts filled with a gray-white tumor measuring 12 × 8 × 6.5 cm. The tumor partially extended into the hilar bile duct, contained abundant gray mucinous material, and had ill-defined boundaries with the surrounding hepatic tissue.

#### Pathology

The primary hepatic lesion was diagnosed as pancreatobiliary-type IPNB complicated by moderately to poorly differentiated invasive adenocarcinoma. The tumor focally invaded the hepatic capsule, and was suspicious for vascular tumor thrombus, although no definite perineural invasion was observed (**Figure 3B**). The resection margin was negative (R0), and the common bile duct margin was also free of tumor. Regional lymph nodes (groups 12 and 13, and para-intestinal nodes) showed reactive hyperplasia only (0/7, 0/3, and 0/6, respectively), indicating no lymph node metastasis (N0). Immunohistochemically, the tumor cells were positive for CK7, CK19, MUC5AC and CEA, and negative for MUC2, CDX2 and CK20. The Ki-67 labeling index was 30%, and p53 staining showed a wild-type pattern (**Supplementary Figure 1B**).

#### Adjuvant therapy decision

Given the high-risk pathological features (moderately to poorly differentiated invasive adenocarcinoma, capsular invasion, and suspected vascular tumor thrombus), adjuvant chemotherapy was strongly recommended according to current guidelines. However, the patient declined all postoperative systemic therapies, including chemotherapy, targeted therapy, and immunotherapy, and was therefore managed with observation alone.

#### Follow-up and secondary pathology

Tumor recurrence was detected 11 months after surgery, accompanied by widespread peritoneal and distant metastases. Resection of metastatic lesions was performed, and pathological examination confirmed metastatic pancreatobiliary-type IPNB (**Figures 4A, B**).

**Figure 4:**
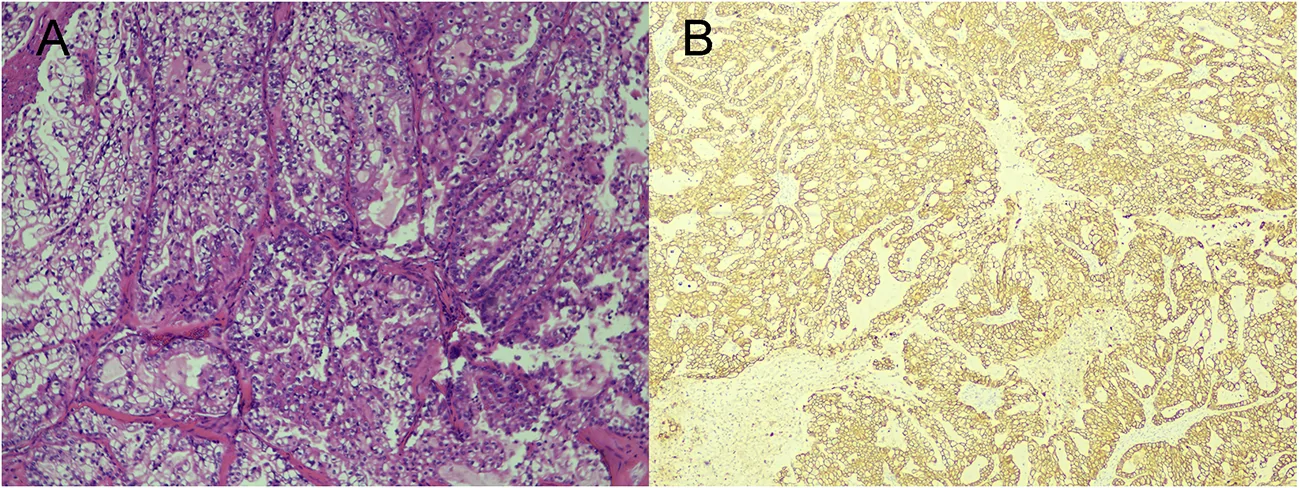
Microscopic features of recurrent bilateral adnexal metastases. **(A)** Low-power H&E staining (×40) shows intraductal papillary proliferation resembling the primary hepatic lesion. **(B)** Tumor cells show strong CK7 immunoreactivity (×40), supporting the metastatic biliary origin of the adnexal masses.

## Discussion

### Possible etiological mechanisms: hepatolithiasis, enterobiliary reflux, and chronic inflammation

The previous biliary-enteric drainage procedures were performed without transection of the common bile duct, leaving a persistent distal blind pouch and resulting in long-term enterobiliary reflux. In the present revision surgery, a standardized biliary reconstruction was performed, which included complete transection of the common bile duct, excision of the previous biliary-enteric anastomotic segment, and re-establishment of bile flow via a Roux-en-Y hepaticojejunostomy. This approach is consistent with current expert consensus, which recommends complete transection of the common bile duct to avoid blind pouch syndrome and to minimize enterobiliary reflux when biliary-enteric reconstruction is indicated (6, 7). In contrast, the previous non-standardized approach, which preserved the native common bile duct, created the anatomical substrate for persistent reflux and chronic inflammation.

Both patients had long-standing hepatolithiasis treated with non-standardized biliary-enteric drainage without complete common bile duct transection. This surgical configuration may lead to a persistent blind pouch and long-term enterobiliary reflux, which in turn may cause recurrent cholangitis and chronic biliary inflammation. Chronic inflammation is a well-recognized contributor to biliary carcinogenesis, and the long interval between the initial drainage procedure and the diagnosis of IPNB in both cases is consistent with a prolonged inflammation-driven process. However, because only two cases were observed and no comparator group was available, these findings cannot establish a causal relationship between non-standardized biliary-enteric drainage and IPNB. The proposed sequence should therefore be regarded as a plausible association rather than an established causal relationship.

### Biological heterogeneity and histological subtypes of IPNB

IPNB is increasingly recognized as a heterogeneous disease entity encompassing intestinal, gastric, pancreatobiliary, and oncocytic subtypes (1, 3, 4, 8, 9). Although subtype classification provides useful morphological stratification, current evidence suggests that prognosis is more closely associated with tumor stage, degree of invasion, anatomical location, multiplicity, and completeness of surgical resection than with histological subtype alone (7, 9–12). Recent type 1/type 2 subclassifications further emphasize architectural complexity, invasion pattern, and clinicopathological behavior rather than mucin phenotype alone (4, 10). Pancreatobiliary-type IPNB is generally regarded as more aggressive at the population level; however, substantial variability exists across all subtypes (3, 4, 9, 10). Therefore, IPNB should be regarded as a spectrum of neoplastic progression rather than a uniform disease. Histological subtype should be interpreted as a tendency rather than a deterministic predictor of clinical behavior.

### Prognostic factors: invasive status, resection margin, and Ki-67

Complete surgical resection remains the standard of care for IPNB. For patients with an R1 margin containing only non-invasive tumor, a conservative approach should only be considered after rigorous multidisciplinary discussion when re-resection is technically challenging. In Case 1, the R1 margin contained only non-invasive papillary tumor, whereas the invasive component was completely resected. Although this patient remained recurrence-free at 12 months, the follow-up period is too short to conclude that residual non-invasive disease has little prognostic impact. Late or metachronous recurrence remains possible, and this observation should therefore be interpreted cautiously and primarily in the context of previous literature rather than as evidence that incomplete resection is acceptable.

The Ki-67 index was assessed by immunohistochemistry. In Case 1, the hotspot method was used according to institutional standard protocols, and the highest value among the examined blocks was recorded; three separate paraffin blocks showed Ki-67 labeling of approximately 70%, 60%, and 60% in hotspot regions, respectively. In contrast, the pathology report for Case 2 documented a Ki-67 labeling index of 30% without explicitly stating whether the hotspot method was used. Therefore, the Ki-67 values from the two cases may not be directly comparable. The higher Ki-67 index in Case 1 (60-70%) than in Case 2 (30%) should not be interpreted as an inverse prognostic marker. Because the Ki-67 labeling in Case 1 was derived predominantly from the high-grade intraepithelial component, whereas the invasive component was minute, this apparent paradox most likely reflects sampling bias and further supports that invasive status, tumor stage, and completeness of resection, rather than overall intraductal proliferative activity, govern clinical behavior (8–12).

Mucin production, including MUC5AC expression as observed in Case 2, has traditionally been associated with earlier clinical detection because intraductal mucin can contribute to intermittent biliary obstruction, upstream ductal dilatation, jaundice, and recurrent cholangitis (3, 4, 11, 13). Nevertheless, this apparent prognostic advantage may partly reflect detection bias rather than intrinsic biological indolence. In the present report, pancreatobiliary-type IPNB with mucinous differentiation was associated with invasive carcinoma and early metastatic dissemination. This finding indicates that mucin phenotype alone is insufficient for prognostication. Instead, mucin expression should be considered a differentiation feature and interpreted in the broader context of tumor stage, invasion, anatomical extent, and imaging findings (11, 13).

### Clinical implications: surgical reconstruction and long-term surveillance

When biliary-enteric reconstruction is otherwise indicated in patients with hepatolithiasis, surgical techniques that avoid a residual blind pouch and minimize enterobiliary reflux should be considered. Complete transection of the extrahepatic bile duct with Roux-en-Y hepaticojejunostomy may be preferred in this setting, although this recommendation is based on pathophysiological reasoning rather than proven preventive efficacy. Patients with a history of non-standardized biliary drainage and long-standing biliary inflammation may warrant long-term surveillance to enable early detection of neoplastic transformation. MRI/MRCP and, when feasible, cholangioscopy are useful for defining the extent of intraductal disease and supporting treatment planning (3, 4, 11, 13, 14). Because biliary carcinogenesis may have a prolonged latency after chronic hepatolithiasis and biliary inflammation, long-term surveillance extending beyond 10 years may be necessary for high-risk patient^s^ (3, 8).

### Strengths and limitations

This report has several strengths. First, it describes two rare cases of IPNB arising after long-standing hepatolithiasis, recurrent cholangitis, and non-standardized biliary-enteric drainage, with a long latency between the initial drainage procedure and the diagnosis of IPNB. Second, detailed surgical and pathological data are provided, including the contrasting clinical courses of Case 1 (R1 resection with residual non-invasive disease but no recurrence at 12 months) and Case 2 (R0 resection but early metastatic recurrence). Third, these cases illustrate the biological heterogeneity of IPNB and support the need for long-term surveillance in patients with long-standing biliary inflammation.

This report also has several limitations. First, only two cases were included, and no comparator group was available; therefore, the observed association between non-standardized biliary-enteric drainage and IPNB cannot establish causality. Second, detailed operative records from the previous drainage procedures were limited, and the exact rationale for preserving the common bile duct could not be definitively determined. Third, follow-up in Case 1 was only 12 months, which is insufficient to assess the long-term prognostic impact of an R1 margin containing non-invasive tumor. Fourth, the Ki-67 values in the two cases were derived from different tumor components and assessed using different methods (hotspot method in Case 1 versus unspecified method in Case 2); moreover, the invasive component in Case 1 was reported only as a microscopic focal lesion without a precise diameter. These factors limit quantitative comparison. Fifth, the decision to decline adjuvant therapy in Case 2 was patient-driven, and its impact on recurrence cannot be determined. Larger studies with standardized reconstruction techniques and long-term follow-up are needed to clarify the relationship between enterobiliary reflux, chronic inflammation, and IPNB development.

## Conclusion

Our findings suggest that non-standardized biliary-enteric drainage with a retained bile duct and blind pouch may be a potential contributing factor to IPNB in patients with hepatolithiasis. Persistent enterobiliary reflux and chronic inflammation may promote a stepwise carcinogenic process, but a causal relationship cannot be established from two cases.

Our cases illustrate the marked biological heterogeneity of IPNB. Complete surgical resection remains the cornerstone of curative treatment; however, the aggressive course in Case 2 despite R0 resection highlights the limitations of resection alone for high-risk invasive disease. This cannot be attributed to the pancreatobiliary-type phenotype alone, but rather likely reflects the adverse pathological features of the tumor, including moderately to poorly differentiated invasive adenocarcinoma, capsular invasion, and suspected vascular tumor thrombus.

Therefore, when biliary-enteric reconstruction is indicated in patients with hepatolithiasis, techniques that avoid a residual blind pouch and minimize enterobiliary reflux should be considered. Long-term surveillance may be warranted for patients with a history of non-standardized biliary drainage and long-standing biliary inflammation.

## Data Availability

The original contributions presented in the study are included in the article/**Supplementary Material**. Further inquiries can be directed to the corresponding author.

## Supplementary material

Supplementary Figure 1 Immunohistochemical profiles of the two IPNB cases (×200). (A) Case 1: CK7(+), MUC5AC(+), MUC2(+), CDX2(+), CK20(+), CEA(+), SATB2(-), Ki-67 labeling index 60%, wild-type p53 pattern. (B) Case 2: CK7(+), CK19(+), MUC5AC(+), CEA(+), MUC2(-), CDX2(-), CK20(-), Ki-67 labeling index 30%, wild-type p53 pattern.

Supplementary Figure 2 Intraoperative view showing a markedly dilated blind jejunal limb.
